# Book Notices

**Published:** 1870-06

**Authors:** 


### Book Notices.

Anatomy, Descriptive and Surgical. By HENRY GRAY, F.R.S., Fellow of the Royal College of Surgeons and Lecturer on Anatomy at St. George's Hospital Medical School. The drawings by H. V. CARTER, M.D., Late Demonstrator of Anatomy at St. George's Hospital. With additional drawings in the second and later editions by Dr. WESTMACOTT. The dissections jointly by the author and Dr. CARTER. With an Introduction on General Anatomy and Development, by T. HOLMES, M.A., Contab.; Surgeon to St. George's Hospital; Mem. Corresp. *De La Soe de Chir. de Paris.* A new American from the fifth and enlarged English edition. With 462 Engravings on wood. Philadelphia: Henry C. Lea. 1870.

As a whole, this is probably the best text-book on anatomy in our language. The present edition is published in good style. For sale by S. C. Griggs & Co., Chicago. Price $7.00.

---

Transactions of the Medical Society of the State of New York, for the year 1869.

This is a well-bound volume of 363 pages, containing, in addition to the record of proceedings at the annual meeting, twenty reports and papers on different topics, biographical sketches of six deceased members, and the lists of permanent and honorary members, etc. It embodies the proceedings and papers of the sixty-second annual meeting of the New York State Medical Society. Copies may be obtained by applying to Wm. H. Bailey, M.D., Secretary, Albany, New York.

---

Our Home Physician: A New and Popular Guide to the Art of Preserving Health and Treating Disease; with Plain Advice for all the Medical and Surgical Emergencies of the Family, etc. By Geo. M. Beard, A.M., M.D.; Lecturer on Nervous Diseases in the University of New York, etc., etc. E. B. Treat & Co., 654 Broadway, New York; C. W. Lilley, 11 Major Block, Chicago.

This is an elegantly bound volume of 1066 pages, intended, as its title imports, to be a popular treatise on medical matters, for use in the family, etc. It is written in good style; and, in most respects, the views presented by the author are judicious. But the single chapter on the use of alcoholic and other stimulants is calculated to do more harm, mentally and physically, than all the rest of the book can do good.

